# Promising Epigenetic Biomarkers for the Early Detection of Colorectal Cancer: A Systematic Review

**DOI:** 10.3390/cancers13194965

**Published:** 2021-10-02

**Authors:** Sorina Andreea Anghel, Corina-Bianca Ioniță-Mîndrican, Ioana Luca, Anca Lucia Pop

**Affiliations:** 1 Department of Clinical Laboratory, Food Safety, “Carol Davila” University of Medicine and Pharmacy, 6 Traian Vuia Street, 020945 Bucharest, Romania; sorina-andreea.anghel@rez.umfcd.ro (S.A.A.); corina-bianca.ionitamindrican@rez.umfcd.ro (C.-B.I.-M.); anca.pop@umfcd.ro (A.L.P.); 2 Department of Molecular Cell Biology, Institute of Biochemistry, Splaiul Independentei 296, 060031 Bucharest, Romania; 3 Department of Toxicology, Faculty of Pharmacy, “Carol Davila” University of Medicine and Pharmacy, 020945 Bucharest, Romania

**Keywords:** colorectal cancer, biomarkers, early detection, NDRG4, BMP3, SDC2, SEPT9, CTCF

## Abstract

**Simple Summary:**

High-performance, non-invasive screening is a requirement in colorectal cancer (CRC) as early detection is a key in reducing disease-related mortality in CRC patients. However, colonoscopy, the actual gold standard in CRC screening, is invasive and often avoided by patients. Conventional screening methods encounter several limitations; therefore, new testing strategies have been considered. DNA methylation is the most prevalent epigenetic alteration that occurs in all stages of carcinogenesis. Our research focused on identifying potential DNA methylation single biomarkers or panels as promising tools in the early detection of CRC; it evaluated methylated genes currently targeted by already approved diagnostic kits. A panel of five CTCF methylated binding sites holds the promise for early-stage specific detection of CRC. CRC screening compliance and accuracy can be enhanced by employing a stool mt-DNA methylation test.

**Abstract:**

In CRC, screening compliance is decreased due to the experienced discomfort associated with colonoscopy, although this method is the gold standard in terms of sensitivity and specificity. Promoter DNA methylation (hypomethylation or hypermethylation) has been linked to all CRC stages. Study objectives: to systematically review the current knowledge on approved biomarkers, reveal new potential ones, and inspect tactics that can improve performance. This research was conducted according to the Preferred Reporting Items for Systematic Reviews and Meta-Analyses (PRISMA) guidelines; the risk of bias was evaluated using the revised Quality Assessment of Diagnostic Accuracy Studies criteria (QUADAS-2). The Web of Science^®^ Core Collection, MEDLINE^®^ and Scopus^®^ databases were searched for original articles published in peer-reviewed journals with the specific keywords “colorectal cancer”, “early detection”, “early-stage colorectal cancer”, “epigenetics”, “biomarkers”, “DNA methylation biomarkers”, “stool or blood or tissue or biopsy”, “NDRG4”, “BMP3”, “SEPT9”, and “SDC2”. Based on eligibility criteria, 74 articles were accepted for analysis. mSDC2 and mSEPT9 were frequently assessed in studies, alone or together as part of the ColoDefense panel test—the latter with the greatest performance. mBMP3 may not be an appropriate marker for detecting CRC. A panel of five methylated binding sites of the CTCF gene holds the promise for early-stage specific detection of CRC. CRC screening compliance and accuracy can be enhanced by employing a stool mt-DNA methylation test.

## 1. Introduction

Since its discovery, cancer has been extensively studied, and insightful physiopathological characteristics are still arising—meanwhile, inadequate invasive screening methods and late diagnostic drive higher mortality. Early detection of precancerous lesions might be promising for prevention, better management, and treatment. Colorectal cancer is the third most common type of cancer in the United States, with a mortality rate of 16.3 and 11.5 for males and females, respectively [1]. Detected at an early stage, the five-year survival rate in CRC is 90%, while for metastatic disease it is 10% [2]. Among the risk factors associated with the onset of colorectal cancer, environmental-related factors contribute significantly, although they can be minimized through lifestyle changes. Besides environmental factors that are considered modifiable, there are a series of non-modifiable risk factors such as age and family or personal history [3].

Moreover, colorectal cancer carcinogenesis is attributed to a series of genetic mutations and epigenetic alterations. Genomic instability has key implications in CRC carcinogenesis through two main pathways: (1) chromosomal instability and (2) microsatellite instability [4]. Regarding epigenetic modifications, the most frequently referred to are (a) DNA methylation, (b) histone modification, and (c) non-coding RNAs [5].

Given the high mortality rates associated with CRC, there is an urgent need for effective screening methods to ensure early detection—the disease being curable if detected in early stages. Currently, the golden standard in CRC screening is colonoscopy. However, colonoscopy has several disadvantages responsible for low compliance despite its high sensitivity (>95%), and its possibility for removing cancerous and precancerous lesions at the time of detection. These limitations include invasiveness, the necessity of bowel preparation, the risk of bowel perforation, and the need for sedation. Other conventional, high-sensitive screening methods—sigmoidoscopy or CT colonography—are semi-invasive tests and display specific limitations [6]. As a non-invasive alternative, the most-used method for CRC screening is fecal occult blood testing (FOBT), through several testing alternatives such as the guaiac fecal occult blood test (gFOBT) or fecal immunochemical test (FIT). Despite the non-invasive character of these screening methods, they have lower sensitivities than a colonoscopy and are mainly used for detecting advanced colorectal neoplasms [7].

**Figure 1:**
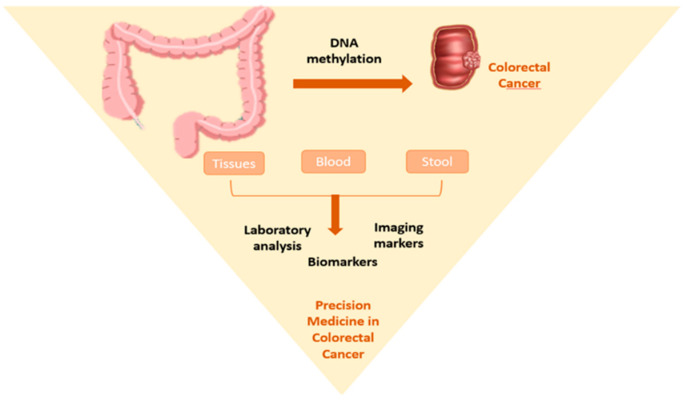
Emerging testing strategies in CRC based on methylated DNA biomarkers.

The above-described limitations of current CRC screening led to the emerging need to develop new, non-invasive methods with high sensitivity and specificity. DNA methylation is a well-known epigenetic modification involved in cancer development and progression. This alteration is responsible for transcriptional silencing of tumor suppressor genes due to the aberrant methylation of 5’-C-phosphate-G-3’ (CpG) islands (CGIs). Based on the possibility of detecting methylated DNA in a wide range of biological samples (blood, tissue, stool) and the fact that these epigenetic alterations occur in the early stages of CRC, identifying DNA methylation-based biomarkers is a valuable tool in the early detection of CRC. Moreover, methylation biomarkers can be used to monitor treatment and prognosis (Figure 1) [8]. 

New testing strategies were considered during the last decade based on DNA methylation, as the most prevalent epigenetic alteration occurring in all stages of carcinogenesis. At present, several screening kits targeting methylated genes have already been approved for use in CRC in certain countries, with acceptable sensitivity and specificity. As higher sensitivity and specificity screening methods are essential for early detection of CRC, other specific methylated genes—potential biomarkers—have been intensely investigated. However, a plethora of research is present on the topic. As the literature lacks systematic research on CRC screening using DNA methylated biomarkers, examining the new biomarkers’ diversity and strength of testing is paramount to further develop optimized and validated DNA methylation tests.

In the present review, we evaluated the performance of methylated genes targeted by already approved diagnostic kits in a few countries worldwide. Subsequently, we focused on identifying new potential DNA methylation single-biomarkers or panels as promising tools in the early detection of CRC. 

## 2. Materials and Methods

### 2.1. Search Strategy

We conducted a systematic literature review exploiting three main electronic libraries: Web of Science^®^ Core Collection (Clarivate Analytics, Philadelphia/London, USA/GB), MEDLINE^®^ (National Library of Medicine’s, Bethesda, MD, USA), and Scopus^®^ (Elsevier, Amsterdam, NL, USA). According to the study protocol (Table S6), the study was conducted based on Preferred Reporting Items for Systematic Reviews and Meta-analyses (PRISMA) [9]. Initial requirements included open access original research published in full-text research articles in peer-reviewed academic journals during the last five years (2017–2021). We used the Boolean operator term AND to focus and narrow the search and the OR term to extend it. 

We searched all fields, topic (Web of Science^®^), article title, abstract, and keywords (Scopus^®^). Firstly, we performed four search queries, one per each investigated biomarker (NDRG4, BMP3, SEPT9, and SDC2), using the key terms: (1) «colorectal cancer» AND « NDRG4» AND « early detection»; (2) «colorectal cancer» AND « BMP3» AND « early detection» (3) « colorectal cancer» AND « SEPT9» AND «early detection» (4) «colorectal cancer» AND «SDC2 » AND «early detection». Then, a broader search was employed: (5) «colorectal cancer» AND «epigenetics» AND «biomarkers»; (6) «colorectal cancer» AND «DNA methylation biomarkers». Finally, a more stage-specific query was addressed: (7) «early-stage colorectal cancer» AND «DNA methylation» AND «stool»; (8) «blood» OR (9) «tissue» OR (10) «biopsy» OR (11) «early detection of colorectal cancer» AND «DNA methylation biomarkers». We selected all randomized clinical trials (RCTs), clinical trials (CT), clinical case series (CCS), clinical cases (CC), reports, and preclinical studies published in the specified period in the searched databases in the English language. There was no restriction on geographical region, ethnicity, or specific population. On 10 July 2021 and 11 July 2021, we searched the Web of Science^®^ Core Collection, and on 24 August 2021, we explored the MEDLINE^®^ and Scopus^®^ databases.

### 2.2. Eligibility 

The included studies reported on early detection biomarkers for primary CRC and complied with the following criteria: (1) DNA methylation markers specific for precancerous lesions and/or CRC Stage 0, I, or II, (2) significant differences between early stage vs. advanced stage CRC, (3) biomarker performance assessment (e.g., sensitivity, specificity), (4) a wide variety of probes and specimens, and (5) any method for methylation status determination.

Furthermore, we excluded doctoral theses, position statements, conference abstracts, editorials, reviews (SR), meta-analyses (MA), or study protocols. We rejected articles containing no screening objective, other cancer types, other epigenetic alteration, other CRC stages (advanced CRC), no DNA methylation biomarker, an analytical approach, treatment evaluation, or no clinical or experimental data. However, we did a snowball search in the MA and SR for relevant original papers that fit our search criteria.

### 2.3. Data Extraction, Analysis, and Quality Assessment

Three authors independently screened and extracted relevant information from the eligible studies, including study population (country, number of subjects, age, presence of histological modification and/or colorectal cancer stage), study design, DNA methylation method, methylation levels, and assay performance in terms of sensitivity and specificity for early detection of colorectal cancer. The studies were qualified for eligibility according to pre-specified inclusion criteria. Any disagreement was discussed and resolved by further analysis and careful examination; a fourth analyst reviewed the investigators’ search steps. Graphs were made using GraphPad Prism^®^ 6.0 (Graphpad^®^ Software Inc., San Diego, CA, USA) and Excel (Microsoft Office^®^, Albuquerque, NM, USA).

The included studies were subject to the quality assessment tool for diagnostic accuracy studies 2 (QUADAS-2) [10]. The tool was modeled for our review topic to determine the risk of bias and concerns regarding applicability over four domains: patient selection, index tests, reference standards, and flow and timing. The risk of bias and applicability concerns were rated as “High”, “Low”, or “Unclear” by two reviewers (S.A.A and C.B.I). Graphical representation and tabular results were made using available online templates from the University of Bristol website. Furthermore, The National Health Medical Research Council (NHMRC) Level of Evidence Hierarchy (2009) was applied to all reviewed studies [11]. 

## 3. Results

### 3.1. Study Selection

**Figure 2:**
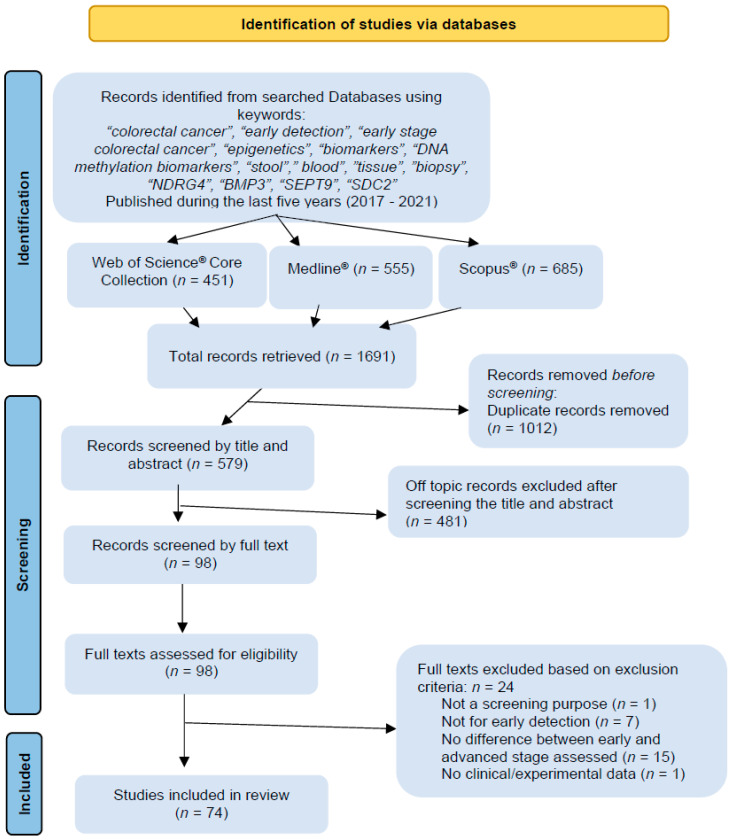
Preferred Reporting Items for Systematic Review and Meta-Analysis (PRISMA) diagram illustrating the literature search process.

The search in the Web of Science^®^ Core Collection database retrieved 451 results, in MEDLINE^®^ 555 results, and Scopus retrieved 685^®^ results, with 1.691 articles over the three databases. After duplicate removal (*n* = 383), 579 articles were screened by title, and if the title was inconclusive, the abstracts were read. After careful selection, 98 articles were chosen for full-text reading. Finally, based on our inclusion and exclusion criteria, 24 articles were eliminated, with 74 articles included in the review based on selection criteria (Figure 2).

### 3.2. Colorectal Cancer, Aging, Epigenetic Clock, and DNA Methylation

The incidence of colorectal cancer increases with age. Biological aging is associated with genomic instability, mitochondrial dysfunction, epigenetic changes, increased cellular vulnerability, and telomeric erosion [12,13].

The rate of CpG site methylation in gene promoters is enhanced with aging. One of CRC’s characteristics is CpG island methylator phenotype (CIMP) which represents the overall hypermethylation status of specific genes that are involved in cellular growth and survival [14]. CIMP displays V-raf murine sarcoma viral oncogene homolog B (BRAF) mutations, methylated mutL homolog 1 (MLH1), and a deficient mismatch repair [15]. With age, methylation of cancer-specific genes occurs in the normal colonic mucosa of healthy subjects with no history or evidence of disease. Therefore, age-related DNA methylation predisposes to increased cancer risk [16]. 

One of the theories of aging proposes the existence of an epigenetic clock that englobes specific methylation signatures that can be predicted and studied through genome-wide techniques. Genomic methylation patterns can serve as biomarkers for biological aging, such that a model of the aging-specific methylome can be developed [14]. Consequently, epigenetic age estimators based on different CpG sites were discovered [17,18,19]. Biological age acceleration, as a difference between epigenetic and chronological age, was correlated with cancer incidence and mortality [20]. DNA methylation age (DNAmAge) is associated with CRC prognosis. Moreover, three algorithms. DNAmMRscore (DNAm mortality risk score), DNAm PhenoAge acceleration (AgeAccelPheno), and DNAm GrimAge acceleration (AgeAccelGrim) can be positively associated with colorectal cancer-specific mortality [21]; blood DNA methylation profiles can be measured using the Infinium Methylation EPIC BeadChip Kit that covers over 850,000 CpG sites (Illumina, Inc, San Diego, CA, USA). Three methylation-based measures of biological aging PhenoAge, GrimAge, and methylation-predicted telomere length were associated with CRC risk and other cancers such as lung, kidney, and urothelial cancer [22]. Furthermore, the epigenetic clock, PhenoAge, is suitable for high CRC risk estimation [23].

Long interspersed nucleotide element-1 (LINE-1) is an autonomous retrotransposon that generates new genomic insertions through the retrotransposition of an RNA intermediate. LINE-1 hypomethylation can serve as a biomarker for human aging [24,25], and is also associated with the early-onset of CRC [26]. The methylation status of DZIP3, an E3 ubiquitin ligase, appears to be the bridge between aging, the immune system, and CRC [13].

### 3.3. Currently Approved and Known Biomarkers; High Performance, Low CRC-Specific Early Detection

Cologuard^®^ is the first FDA-approved stool DNA test for average-risk CRC screening (in 2014)—a multi-target stool DNA (mt-sDNA) test with high sensitivity which analyzes several molecular markers, including N-Myc Downstream-Regulated Gene 4 Protein (NDRG4) and Bone Morphogenetic Protein 3 (BMP3) methylation status, KRAS mutation and β-actin, combined with FIT for CRC screening. Compared to other screening methods based on fecal hemoglobin detection as a single marker, the mt-sDNA test also detects mutations and DNA methylation in all stages of carcinogenesis, from premalignancy to advanced stages. In contrast, the detection of fecal hemoglobin alone is more reliable in the late stages, given the fact that precancerous lesions may not bleed [27,28].

**Figure 3:**
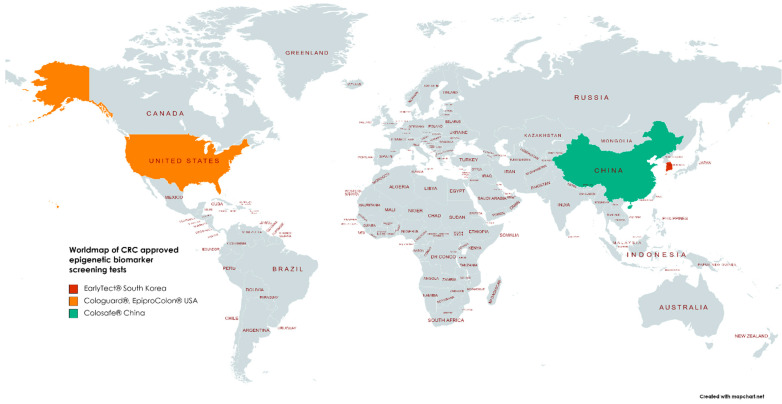
Map of approved epigenetic screening test kits Cologuard^®^, Epi proColon^®^—USA, Colosafe^®^ China, EarlyTect^®^ Korea. Colodefense^®^ (China, 2021) is used at present only for research objectives.

Epi proColon^®^ is the first blood-based screening test approved by the FDA, in 2016. The test consists of a qualitative analysis based on PCR detection of methylated SEPT9 (septin 9). Methylated SEPT9 levels are significantly elevated in both tissue and blood samples from patients with CRC compared to healthy individuals, having high specificity but a lower sensitivity compared to Cologuard^®^ and other screening methods [29]. Besides Cologuard^®^ and Epi proColon^®^, single-target stool DNA tests are also available for CRC screening. EarlyTect™-Colon Cancer (Genomictree, South Korea) approved by the Korean [30], and Colosafe^®^ (Creative Biosciences China), approved by the China National Medical Products Administration are two st-sDNAs [31,32] (Table 1, Figure 3).

We examined the last five years’ literature for methylated genes currently targeted by FDA or NMPA screening kits: mSDC2, mSEPT9, mNDRG4, and mBMP3. With our specific filters, we found six studies dedicated to mSDC2 [33,34,35,36,37,38] and seven studies for mSEPT9 [39,40,41,42,43,44,45]; two described mBMP3 performance [46,47], while no study evaluated mNDRG4 alone. A multi-target approach evaluating these biomarkers and others was employed in 16 studies, 5 of them deciphering mSDC2 and mSEPT9 performance [48,49,50,51,52].

#### 3.3.1. Syndecan-2 (SDC2)

SDC2 is a transmembrane protein implicated in cellular proliferation, migration, cell–matrix interaction, and angiogenesis [53]. SDC2 methylation (Table S1) was quantified in two studies using tissue and stool [33,34]; one study used tissue and bowel lavage [35], and three studies evaluated DNA methylation only in stool [36,37,38]. Only one study had paired samples [33]. Sensitivities for stages I/II were between 83.3–91.4%, and 89.6–100% for stage III/IV [34,36,37]. Although evaluation of SDC2 methylation is indicated for early detection of colorectal cancer, it seems that assay sensitivity is superior for stage III/IV, while for stage I/II is satisfactory, thus displaying a limited stage specificity. 

#### 3.3.2. Septin 9 (SEPT9)

SEPT9 belongs to the septin family, being a cytoskeleton component with GTP-binding protein activity. Therefore, it has a role in essential processes, including cell division. CpG island hypermethylation of the SEPT9 promoter leads to altered expression—eliminating its tumor suppressor activity, which contributes to carcinogenesis [54]. mSEPT9 was evaluated in four studies as a single biomarker [39,40,42,45]. Three studies compared mSEPT9 performance with other known screening tools [41,43,44] (Table S2). Plasma was used in six studies [39,40,41,42,43,44], while one employed a paired sample approach investigating plasma and stool samples from the same patients [45]. Epi proColon^®^ was frequently used for plasma mSEPT9 measurement. Methylated SEPT9 in plasma samples had a higher sensitivity when compared with FOBT or known tumor protein markers—CEA and CA19-9. When combined, FOBT and mSEPT9 assessment led to maximum sensitivity (100%) for stage I CRC [41]. Furthermore, sensitivity is higher for mSEPT9 in stool samples vs. plasma samples while having a similar specificity [45]. Some studies also evaluated mSEPT9 prognosis significance (extensively reviewed here [55]) and its role in recurrence monitoring [40,42,43,44].

#### 3.3.3. Bone Morphogenetic Protein (BMP3)

Bone morphogenetic protein (BMP3) is secreted by osteoblasts and osteocytes, contributing to bone mass regulation [56] and having an essential role in cellular development and growth. Furthermore, BMP3 promoter hypermethylation induces its inactivation with subsequent tumorigenesis implications [57]. In both studies analyzed, BMP3 was evaluated in an Iranian population; the methylation status was highest in stage IIA CRC (66.6%), attributing a role to it in early detection [46]—although both studies showed a decrease in overall sensitivity and specificity. Furthermore, the authors concluded that BMP3 performance is not sufficient for use as a single biomarker; instead, it can be assessed with other specific methylated genes [46,47].

#### 3.3.4. N-Myc Downstream-Regulated Gene 4 (NDRG4)

N-Myc Downstream-Regulated Gene 4 (NDRG4), expressed within nervous system structures throughout the body, but predominantly studied in the brain and heart, was proven to be expressed explicitly in enteric neurons in a study using tissues from NDRG4 wild-type, heterozygous, and knockout mice and humans; immunoreactivity was restricted to the enteric nervous system (ENS) [58]. A recent study aiming to identify whether the ENS, via NDRG4, affects intestinal tumorigenesis showed that Ndrg4 knockdown in CRC models and in an indirect co-culture of primary enteric nervous system (ENS) cells was associated with enlarged intestinal adenoma development—the organoid growth being boosted by the Ndrg4−/− ENS cell secretome—the ENS, via loss of Ndrg4, being involved in colorectal pathogenesis [59].

The reduction of ENS plexus size is accompanied by the increased number of galanin-immunoreactive neurons; the neuroprotective peptide galanin may inhibit the extrinsic pathway of apoptosis, and in this way promote (colon) cancer cell survival [60].

#### 3.3.5. Methylated Genes Panel Containing Approved Biomarkers

We considered a biomarker panel any evaluation of two or more methylated genes. Most studies described ColoDefense^®^, a yet unapproved test for clinical diagnosis, which combines mSDC2 and mSEPT9 detection [48,49,50,51,52] (Table S3). For stage I CRC, the ColoDefense^®^ test showed a sensitivity between 69.2–81.8%, stage II CRC 85.7–100%, stage III 88.9–89.7%, and stage IV 75–100%. The maximum specificity was 93.2%. Besides the performance evaluation of the blood ColoDefense^®^ test, two articles had a more particular approach: (a) testing protein biomarkers concomitant with methylated SDC2 and SEPT9 [51] or (b) evaluating a possible false-positive result based on leukocyte genomic DNA [52]. Comparing sensitivities for methylated genes vs. methylated genes plus tumor-specific protein markers (CEA, AFP, CA19-9), an increase was observed (from 35.3 to 47.1% for stage 0+I, 48.6 to 74.3% for stage II, 64.0 to 80% for stage III, and 89.7 to 96.6% for stage IV) [51]. As for point (b), when mSEPT9 and mSDC2 were assessed from blood leukocytes, no significant difference was noticed between normal subjects vs. individuals with colorectal cancer (stages I-IV) [52]. At the same time, the ColoDefense test could differentiate CRC cases from precancerous lesions or healthy individuals [52].

Assessing DNA methylation biomarkers together with mutated ones is not uncommon. Three studies that evaluated mt-DNA tests used the same strategy as the Cologuard^®^ test. A mNDRG4, mBMP3, and KRAS mutations, and a hemoglobin test, evaluated in a screening setting, could distinguish between different precancerous lesions and be more sensitive than FIT in detecting them [61]. A multi-faceted stool-based assay that covered three methylated biomarkers (SEPT9, NDRG4, BMP3), three mutated genes (KRAS, BRAF, PI3KCA), FIT and a bacteria level measurement of *Fusobacterium nucleatum* and *Parvimonas micra,* generated an increase in CRC detection rates from stage I to stage III, followed by a decrease in stage IV [62]. Additionally, KRAS mutations were explored together with mNDRG4, methylated tissue factor pathway inhibitor 2 (mTFPI2), and mSDC2 in an mt-DNA assay which exceeded FOBT in detecting stage I-III CRC [63].

Combined mSDC2 and mTFPI2 measurements showed a higher sensitivity for identifying adenoma and CRC. Moreover, methylation determination in stool probes outperformed FOBT and protein markers [64]. When a panel containing mSDC2, methylated secreted frizzled-related protein 1 (mSFRP1), methylated secreted frizzled-related protein 2 (mSFRP2) and methylated proline-rich membrane anchor 1 (mPRIMA1) was investigated for detecting adenoma and CRC in plasma, a higher performance was obtained compared with each methylated gene alone [65]. Furthermore, mSDC2 and mSFRP2 can be simultaneously measured with a novel test—SpecColon—which shows an increase in sensitivity with CRC stage and is convenient to perform in advanced adenoma [66].

Multiplexing SEPT9 with O-6-methylguanine-DNA methyltransferase (MGMT) and Ras association domain family 1—isoform A (RASSF1A) evidences an increase in sensitivity with a decrease in specificity for CRC. As for stages I and II, a sensitivity of 100% is reached, pointing to a key role in early detection [67]. A developed model comprising of SDC2, SEPT9, and five other hypermethylated gene promoter regions — homeobox protein aristaless-like 4 (ALX4), BMP3, Neuronal Pentraxin 2 (NPTX2), Retinoic Acid Receptor Beta (RARB), and vimentin (VIM)—displayed an early-stage specific performance [68]. A panel of 80 methylation markers including SEPT9, IKAROS family zinc finger 1 (IFZF1), Branched chain amino acid transaminase 1 (BCAT1), vimentin (VIM), and others, displayed a sensitivity of 74% and a specificity of 90% for identifying early-stage CRC [69].

NDRG4, BMP3, SEPT9, and SDC2 were evaluated in two studies [70,71]. The former study found that mBMP3 detection is unsuitable for colorectal cancer diagnostics due to its decreased methylation frequency in tissue or stool samples [70]. However, the latter showed an improved performance by exploiting a merged assay in stool samples by sDNA-FOBT (DNA methylation markers BMP3, NDRG4, SDC2 together with FOBT) [71].

### 3.4. Promising Biomarkers in Early Screening of Colorectal Cancer, a Step Forward towards a Precision Medicine Approach

Several studies investigated potential new biomarkers in the early screening of colorectal cancer. As single biomarkers, CLIP4 was evaluated in one study [72], TWIST1 in three articles [73,74,75], and LINE-1 in two [76,77]. A panel of methylated biomarkers was also employed—C9orf50 was assessed together with CLIP4 and KCNQ5 [78], with TWIST1 [79] or just with KCNQ5 [80]. Other potential biomarkers and their performance are displayed in Table S4 [81,82,83,84,85,86,87,88,89,90].

#### 3.4.1. CAP-Gly Domain Containing Linker Protein Family Member 4 (CLIP4)

**Figure 4:**
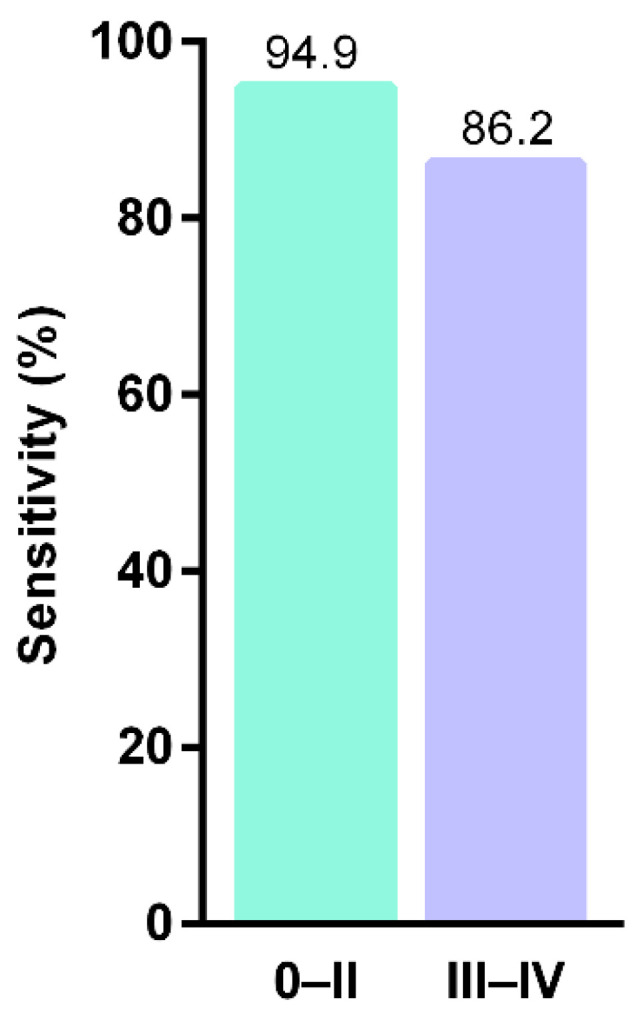
Sensitivities of stool mCLIP4 test in detecting different CRC stages (after Cao et al., 2021) [72].

The mammalian cytoplasmic linker protein (CLIP)-170 (CLIP 1-4) links the microtubule plus ends to kinomeres, endocytosis vesicles, and the steering brink of migrating cells. Recent studies have shown that methylated CAP-Gly domain containing linker protein family member 4—CLIP4, may be a potential biomarker for early detection of CRC. An mCLIP4 assay was carried out on stool and tissue samples, showing a sensitivity of 90.3% and specificity of 88.4% (Figure 4) [72,78].

#### 3.4.2. Alpha 1-Antitrypsin (A1AT)

Another study highlights alpha 1-antitrypsin (A1AT) as a potential biomarker in colorectal cancer detection. Plasma concentrations were measured for both CEA and A1AT, in healthy and CRC patients. Plasma concentrations of A1AT were correlated with tumor stage, and A1AT determination had better sensitivity and specificity than CEA for early detection of CRC. A1AT is highly expressed in inflammation, mainly when localized at hepatocytes. This gene is also expressed in colonic tumor cell lines. Some studies have suggested an increase in A1T1 in cancers such as those of the pancreas, breast, and liver [91].

#### 3.4.3. Zinc-Finger Protein CCCTC-Binding Factor (CTCF)

**Figure 5:**
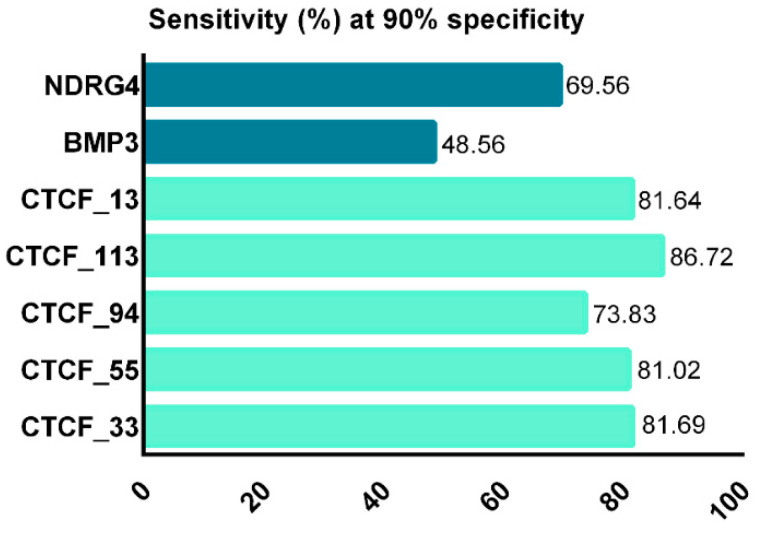
Comparison of CTCF-binding site markers with existing markers (after Liu et al., 2017) [92].

The five potential zinc-finger protein CCCTC-binding factors function as transcriptional activator, repressor, or insulator proteins; CTCF_33, CTCF_55, CTCF_94, CTCF_113, and CTCF_13 biomarkers analyzed in a study from China showed better performance than two well-known methylation biomarkers, NDRG4 and BMP3. Furthermore, with a sensitivity of 93.54% and a specificity of 94.05% (Figure 5), CTCF-binding sites may be possible biomarkers in colorectal cancer detection [92].

#### 3.4.4. Metallophosphoesterase Domain Containing 2 (MPPED2) Gene

The MPPED2 gene regulates many cellular functions, including differentiation, proliferation, and cellular apoptosis—well known for the link between their malfunctioning and cancer pathogenicity. Gu et al. indicate that epigenetic changes in the MPPED2 promoter region are encountered during colorectal cancer. The study included (a) a group with colorectal tissue lesions, (b) a group with polyps, and (c) a group with adenoma, (d) primary carcinomas, and (e) normal tissues. The study results indicated that hypermethylation of MPPED2 promoter is common in CRC. The methylation state of MPPED2 showed an exponentially increase between colorectal lesions groups from 5.58% (polyps) to 14.5% (adenoma) and 30.56% (carcinoma). Taking these results into account, methylated MPPED2 could be a promising biomarker for the early diagnosis of CRC [93].

#### 3.4.5. Smooth Muscle Protein 22α (SM22α)

The link between protein expression and the methylation status of the smooth muscle protein 22α (SM22α) was investigated in 78 cases of CRC. The results were promising, showing that the methylation level of the SM22α promoter is higher in CRC tissues compared to control samples. Furthermore, hypermethylation of the SM22α gene may occur in the early stages of CRC, so it may be a biomarker in diagnosis of CRC [94].

#### 3.4.6. Transmembrane Protein 240 (TMEM240)

To date, the role of TMEM240 in cancer pathogenicity has not been completely elucidated. Chang et al. investigated the relationship between expression and methylation status with CRC. Hypermethylation of TMEM240 in tumor tissues was increased compared to normal tissues. Low expression of the TMEM240 protein has been observed in about half of patients with inflammatory bowel diseases, which have a three to five times higher risk of developing colorectal cancer. The study’s results showed that hypermethylation and low expression of TMEM240 are potential biomarkers for colorectal cancer detection, poor prognosis, and early recurrence prediction [95].

#### 3.4.7. Potassium Calcium-Activated Channel Subfamily M Alpha 1 (KCNMA1)

KCNMA1 encodes the α-subunit of the large conductance, voltage, and Ca2+-activated (BK) potassium channel; it is widely distributed across tissues, including both excitable and non-excitable cells [96]. Basie et al. have extensively studied the expression of the KCNMA1 gene associated with colorectal cancer. It has been observed that in patients with colorectal cancer, levels of KCNMA1 are considerably low due to methylation, without being able to distinguish between cancer stages. Therefore, some validation studies may be required on the use of KCNMA1 as a biomarker in detecting CRC [97].

#### 3.4.8. Long Interspersed Nuclear Element-1 (LINE-1)

Current studies have shown that analysis of LINE-1 methylation levels in circulatory DNA could discriminate lung cancer patients from patients with chronic inflammatory lung diseases [98]. Therefore, the assessment of methylated LINE-1 may serve as a valuable tool for cancer screening. Two studies also evaluated hypomethylation of long interspersed nuclear element-1 (LINE-1) as a potent early detection biomarker in CRC [76,77]. Measurement of cell-free DNA long interspersed nuclear element-1 hypomethylation index (cfDNA LHI) was achieved with 63.2% sensitivity and 90% specificity for early-stage I/II CRC [76]. In addition, LHI can differentiate between types of histological alteration by having a directly proportional relationship with malignancy degree [77]. Values of LHI increase in the following order: healthy subjects, non-advanced adenoma (NAA), advanced adenoma (AA), adenocarcinoma (AC) [77].

#### 3.4.9. Other Potential Biomarkers in Literature

In 2019, a study correlated the link between androgen receptor AR () and colorectal cancer risk in 378 patients. AR hypomethylation in young patients (under the age of 60) increases the risk of CRC. The explanation may be that in young patients, androgen levels are higher, the occurrence of hypomethylated AR is higher, and thus the risk of CRC increases [99]. Glycoprotein nmb (GPNMB) was analyzed from cancerous tissue samples (*n* = 20), non-advanced adenoma tissues (*n* = 21), advanced adenoma (*n* = 48), and normal tissue (*n* = 20). The methylation status of the GPNMB gene can be used to track the progression of colorectal lesions [100]. Hypermethylation of three genes HOXA2, HOXA5, and HOXA6 is detected in CRC patients, with HOXA5 having the highest methylation status [101].

In CRC, Glutamate Ionotropic Receptor AMPA Type Subunit 4 (GRIA4), Solute Carrier Family 8 Member A1 (SLC8A1), and Synapsin III (SYN3) have a higher methylation degree in tissue, stool, and cfDNA [102]. Methylated Solute Carrier Family 30 Member 10 (SLC30A10), claudin 1 CLDN1, and Inhibin Subunit Beta A (INHBA) were evidenced by SureSelectXT Methyl-Seq as being able to differentiate between normal and tumor tissue [103]. Two CpG sites in the promoter region of KIAA1549L are hypermethylated in CRC, pointing to a potential early detection specific biomarker [104]. Also, two CpGs (cg09239744 and cg12587766) may be used for diagnostic CRC [105]. The age of CRC onset has decreased a lot over the last decade. Hence, an increased survival rate can be reached by early diagnosis and prompt action. Recent studies focused on potential biomarkers that can be used in medical practice for the early detection of CRC, which may include the above-mentioned genes or others such as UNC5D, KCNA1 [106], FMN2 [107], JAM3 [108], GSDME [109], CRF [110], SMAD3 [111], SCTR [112], CNRIP1 [113], NEUROG1 [114], and p16 [115].

#### 3.4.10. Quality Assessment of the Included Studies

**Figure 6:**
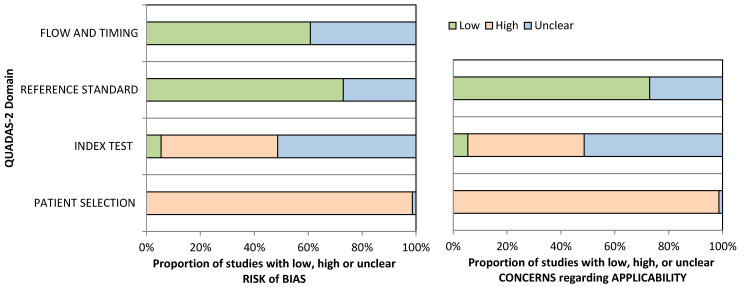
QUADAS-2 quality assessment overview for the included studies.

Quality assessment results using the QUADAS-2 tool are shown below as a summary graph (Figure 6), and in Table S5, tabular results are presented. The patient selection domain shows the highest potential risk of bias due to the case–control study design. The index test rated unclear risk of bias in 39/74 studies, followed by 32 studies which were scored as having a high potential risk of bias considering their ambiguity or lack of information regarding blinding, performing the test as per the manufacturer’s guidelines, or setting a cut-off value. For most of the studies (54/74), if colonoscopy was used as the reference standard, the risk of bias was considered low, while an unclear rating was giving for any uncertainty concerning colonoscopy assessment prior to methylation assays. The flow and timing domain was judged as having a low risk of bias in 45 studies. Applicability concerns were serious for patient selection because included studies employed a clinical setting rather than a screening one.

**Figure 7:**
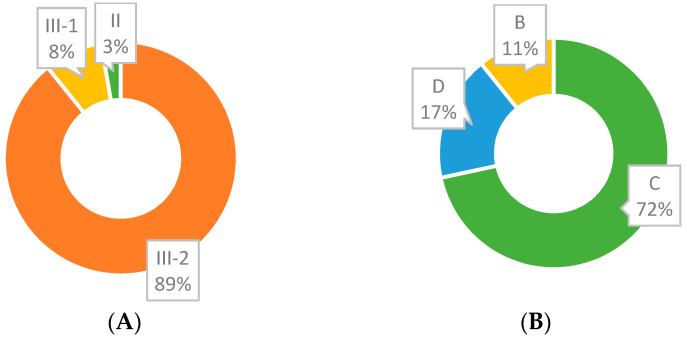
(**A**). III-2 accounts for the majority of ’Evidence for Outcome—Level (Quality) of evidence for diagnostic tests” (NHMRC Evidence Hierarchy). (**B**) C accounts for the majority of grades (Strength) of recommendation.

Level of evidence and grade of recommendation for each included study are shown in Table S5. As already mentioned, our database search did not retrieve RCTs, but case–control studies. Based on the NHMRC Evidence Hierarchy, a level of evidence of III-2 was observed in 66/74 studies (Figure 7A) due to lack of an independent, blinded comparison between methylated DNA test and colonoscopy, as it has not been employed or reported. Only two studies were classified as evidence level II. As for grade of recommendation, C was scored frequently (53/74; Figure 7B), in accordance with the bias assessment and evidence level of reviewed studies.

## 4. Discussion

Cancer is considered a genomic disease with complex occurrence mechanisms, such as defective cellular apoptosis, which causes uncontrolled cell proliferation. Moreover, in the case of colorectal cancers, each tumor has an unique genetic signature. Specific cancer genes are brought to the fore: oncogenes (these genes are encoded by alterations) and tumor suppressor genes (these genes are inactivated in tumorigenesis). Oncogenes can encode factors that influence cell survival and proliferation. In contrast, tumor suppressor genes limit proliferation, growth, motility, and cell invasion. The inactivation of tumor suppressor genes can occur by mutations and promoter methylation [116].

The molecular hallmarks of CRC are microsatellite instability (MSI), chromosomal instability, and CpG island methylator phenotype (CIMP). In the path of chromosomal instability an activation of the KRAS and BRAF genes occurs, along with an inactivation of the tumor suppressor genes, TP53 and APC, and loss of heterozygosity for the long arm of chromosome 18. All these sub-cellular events generate the phenotypically—healthy genetically—mutated cells to transform into cancer cells. The TP53 gene, called the genome guard, has a role in senescence, cellular apoptosis, and DNA repair. In more than 50% of colorectal cancer cases, the TP53 gene is mutated. Fearon and Vogelstein proposed a model in which the suppressor genes APC, DCC, p53, and oncogene KRAS are involved. Chromosomal changes involve the arms of the 8p, 5q, 18q, and 17p chromosomes. In the first stage, the inactivation of the APC gene led to the appearance of adenoma in the normal colon mucosa; the growth of adenoma is correlated with the appearance of KRAS mutations, localized on the chromosome 12p, subsequently resulting in genetic changes—especially deletions of the genes on chromosome 18q. DCC is located on chromosome 18q; approximately 70% of colorectal cancer cases predicted allelic losses of DCC. In the Fearon and Vogelstein model, in the process of transition from adenoma to cancer, mutation or loss of p53 on 17p appeared [117].

A typical path in sporadic colorectal cancers is the microsatellite instability (MSI) pathway which involves a huge accumulation of mutations. MSI occurs due to an inactivation/disturbance at the level of the DNA replication error repair system (MMR), which is responsible for supervising and correcting errors introduced in microsatellites. Microsatellites are repetitive sequences of DNA; fluctuations in the length of the microsatellite, called instability, can mean that the genes repairing replication errors do not work correctly. Faulty repair of replication errors is mainly caused by a methylation anomaly of the MLH1 replication error repair gene, being a sporadic process without the involvement of heredity. Another mechanism may be a mutation of a hereditary nature, if the error-repair genes (MLH1, MSH2, MSH6) produce a genetic predisposition to CRC [118].

Our study evaluated the currently used methylated genes’ performance and identified various potential biomarkers that can be used for early diagnosis. Approved test kits targeting epigenetic markers can be improved using a merged assay (a molecular one, such as SDC2 with SEPT9 plus a human hemoglobin test, for example). Cologuard^®^ already uses this approach, but taking into account that it was the first FDA-approved DNA stool test, it has a decreased performance (compared with the golden standard)—mainly because of methylated NDRG4 and BMP3. BMP3 performance is not satisfactory for use as a single biomarker [46,47]. Furthermore, its methylation levels were reduced or even undetectable in CRC patients, further reinforcing the examination of current data [70].

Using multiple markers or a methylation panel for screening may improve specific characteristics of non-invasive screening procedures. For example, multiplex detection of mSDC2 and mSEPT9 (Colodefense^®^ test) shows high sensitivity and specificity compared with each marker alone, independently of the CRC stage. Nevertheless, the methylation status of currently used biomarkers increases with tumor size or disease progression, making them suitable for screening, diagnostics, and prognostics, but not necessarily for CRC early detection specifically. In contrast, methylated CTCF-binding sites showed an increased accuracy for detecting early-stage CRC. In addition, the assessment of each mCTCF-binding site exhibited higher sensitivity and specificity for stage I-II than for terminal stages. Moreover, when a panel of five mCTCF-binding sites was examined, a sensitivity of 91.67% and a specificity of 94.05% were recorded for adenomas, pointing towards a more precise early-stage biomarker [92].

A potential misinterpretation of CRC screening results may arise from sample type. Tests for detecting DNA methylation can be performed on tissue, blood, stool, or even urine. Stool DNA represents the sample of choice for CRC screening because tumor-circulating DNA (ctDNA) originates directly from the tumor tissue, while ctDNA from plasma may degrade over time [119]. As reported, mSEPT9 was evaluated from stool and plasma samples, the latter determination leading to a lower value that does not accurately reflect SEPT9 methylation grade [45].

Most studies reviewed here used qMSP (quantitative methylation-specific PCR) for DNA methylation measurement, bisulfite pyrosequencing, or LTE-qMSP. PCR data analysis is dependent on the chosen algorithm, which dictates the balance between sensitivity or specificity. It is essential to exclude negative subjects for early detection, implying high specificity as a requirement and assaying more than one PCR replicate [120]. Articles included in this study followed different algorithms, explaining the observed differences in assay performance for the same biomarker. A more standardized, precise approach is needed to overcome these dissimilarities.

Our study has some limitations. Firstly, we systematically searched three databases, none of which screened running clinical trials. We did not searched all available libraries, thus a probability of missing some relevant studies exists. Some studies were excluded because performance parameters were not reported, and we did not have the appropriate tools to calculate them. We excluded research that investigated several types of cancer simultaneously, including CRC, as not being exclusively focused on CRC screening; future research may focus on multiple-target cancer screening using epigenetic biomarkers. Studies reviewed here usually followed a case–control design with inferior strength due to the low sample size and overall methodology. Additionally, a more robust statistical approach could not be addressed because of data heterogeneity.

## 5. Conclusions

This study offers a valuable information source for further investigation and exploration of early detection biomarkers for CRC. We displayed approved markers’ performances using a gene-specific methylation approach and discussed potential screening biomarkers (CLIP4, A1AT, MPPED2, C9orf50, KCNQ5, and others), covering the last five years in the literature. Moreover, we could not emphasize enough the need for updates in present CRC screening guidelines regarding non-invasive methods.

## Figures and Tables

**Table 1:** List of approved CRC screening epigenetic biomarkers tests.

Test	Year	Sample	Biomarker Target
ColoGuard	2014	Stool	NDRG4, BMP3, KRAS, β-actin
Epi proColon^®^	2016	Blood	SEPT9
EarlyTect™-Colon Cancer	2018	Stool	SDC2
Colosafe^®^		Stool	SDC2

## Supplementary Materials

The following are available online at https://www.mdpi.com/article/10.3390/cancers13194965/s1, Table S1: SDC2 performance in multiple studies, Table S2: mSEPT9 performance comparison with other screening methods, Table S3: SEPT9 and SDC2 (Colodefense^®^ test) performance in multiple studies, Table S4: Potential biomarker performance in multiple studies, Table S5: Quality assessments of studies. QUADAS2-LoE-GoR, Table S6: Research strategy (Annex 1).

## Abbreviations

5hmC	5-Hydroxymethylcytosine
A1AT	Alpha 1-antitrypsin
AA	Advanced adenoma
ADHFE1	Alcohol Dehydrogenase Iron Containing 1
AFP	Alpha-fetoprotein
AR	Androgen Receptor
BMP3	Bone Morphogenetic Protein 3
C9orf50	Chromosome 9 Open Reading Frame 50
CA19-9	Carbohydrate antigen 19-9
CCCTC	Zinc-finger protein CCCTC-binding factor
CEA	Carcinoembryonic Antigen
CHST11	Carbohydrate Sulfotransferase 11
CI	Confidence Interval
CIMP	CpG island methylator phenotype
CLDN1	Claudin 1
CLIP4	CAP-Gly Domain Containing Linker Protein Family Member 4
CNRIP1	Cannabinoid Receptor Interacting Protein 1
CRC	Colorectal cancer
CRF1	Corticotropin-Releasing Factor
CTCF	CCCTC-Binding Factor
DCC	DCC Netrin 1 Receptor
FIT	Fecal immunochemical test
FMN2	Formin 2
FOBT	Fecal occult blood testing
GDNF	Glial Cell-Derived Neurotrophic Factor
GPNMB	Glycoprotein Nmb
GRIA4	Glutamate Ionotropic Receptor AMPA Type Subunit 4
GSDME	Gasdermin E
HAND2	Heart And Neural Crest Derivatives Expressed 2
HGA	High-grade adenoma
HOXA 2	Homebox A2
HOXA 5	Homebox A5
HOXA 6	Homebox A6
HP	Hyperplastic polyps
INHBA	Inhibin Subunit Beta A
JAM3	Junctional Adhesion Molecule 3
KCNA1	Potassium Voltage-Gated Channel Subfamily A Member 1
KCNMA1	Potassium Calcium-Activated Channel Subfamily M Alpha 1
KCNQ5	Potassium Voltage-Gated Channel Subfamily Q Member 5
KIAA1549L	KIAA1549 Like
KRAS	KRAS Proto-Oncogene
LGA	Low-grade adenoma
LINE-1	Long interspersed nuclear element-1 (LINE-1)
MA	Meta-analyses
MLH1	MutL Homolog 1
MMD	Dysplasia of mild and moderate degrees.
MPPED2	Metallophosphoesterase Domain Containing 2
MSH2	MutS Homolog 2
MSH6	MutS Homolog 6
NAA	Non-advanced adenoma
NHMRC	National Health and Medical Research Council Evidence Hierarchy
NDRG4	N-Myc downstream-regulated gene 4
NEUROG1	Neurogenin 1
PI3KCA	Phosphatidylinositol-4,5-Bisphosphate 3-Kinase Catalytic Subunit Alpha
PRIMA1	Proline-rich membrane anchor 1
QUADAS-2	Quality Assessment of Diagnostic Accuracy Studies criteria
SCTR	Secretin Receptor
SDC2	Syndecan 2
SEPT9	Septin 9
SFRP2	Secreted frizzled-related protein 2
SLC30A10	Solute Carrier Family 30 Member 10
SLC35F3	Solute Carrier Family 35 Member F3
SLC8A1	Solute Carrier Family 8 Member A1
SM22α	Smooth muscle protein 22α
SMAD3	SMAD Family Member 3
SNAP91	Synaptosome Associated Protein 91
SORCS1	Sortilin Related VPS10 Domain Containing Receptor 1
SP	Small polyps
SYN3	Synapsin III
TFPI2	Tissue factor pathway inhibitor 2
TMEM240	Transmembrane Protein 240
TP53	Tumor Protein P53
TWIST 1	Twist Family BHLH Transcription Factor 1
UNC5D	Unc-5 Netrin Receptor D
VIPR2	Vasoactive Intestinal Peptide Receptor 2
