# Supplementary material for: Promising Epigenetic Biomarkers for the Early Detection of Colorectal Cancer: A Systematic Review
Source: Cancers (Basel). 2021 Oct 2;13(19):4965. doi: 10.3390/cancers13194965 (PMC8508438; doi:10.3390/cancers13194965)
Supplement: Supplementary file 1 [file cancers-13-04965-s001.zip › Supplementary Table S6 Research strategy.pdf]

Annex 1

Systematic Review Protocol & Support Template

1. **Title of the review**	Promising epigenetic biomarkers for the early detection of colorectal cancer: a systematic review
First reviewer	Pharm. Andreea Sorina Anghel (ASA)
Team of reviewers	Pharm. Ioana Luca (IL)<br>Drd. Pharm Corina Bianca Ionita-Mindrican (CBIM)
Supervisor/Project PI	Dr. Anca Lucia Pop (ALP)

2. **SR overview**	Advice sought from ALP
Protocol development	
Literature searching	Already had training from library on literature searching and EndNote and also did literature review for PhD study (ASA, IL, CBIM)
Quality appraisal	Advice gained from ALP and from reading around the area
Data Extraction	Advice gained from ALP
Synthesis	Advice gained from ALP

3. **Background**	There is unreviewed data on epigenetic biomarkers in CRC and a plethora of studies
**Specific objectives**	DNA methylation single-biomarkers or panels as approved tools in the early detection of CRC
	Identification of potential new DNA methylation single-biomarkers or panels as promising tools in the early detection of CRC

4. **Search methods**	
Electronic databases Please list all databases that are to be searched and include the interface (eg NHS, EBSCO, etc) and date ranges searched for eac	5. Web of Science Core Collection<br>6. PUBMED/MEDLINE<br>7. SCOPUS<br>past five years for each
Other methods used for identifying relevant research (ie contacting experts and reference checking)	Reference checking, reverse / hand searching of these.<br>Identifying possible data from conferences attended

5. **Methods of review**	
**Details of methods**<br>Number of reviewers, how agreements to be reached and disagreements dealt with, etc.	Two main reviewers and a third to resolve any disagreements<br>Main reviewers Agree data to be extracted and terminology used in CPD to be clarified before hand

**Quality assessment**<br>Tools or checklists used with references or URLs	Protocol will define the method of literature critique/ appraisal use, and will use STROBE tool for relevant content and methodology used in the each of the papers to be reviewed
**Data extraction**<br>What information is to be collected on each included study. If databases or forms on Word or Excel are used and how this is recorded and by how many reviewers	Data extraction form in Word document<br>EndNote to be used to keep track of references<br>Reviewer number 1 (ASA) will review first, followed by reviewer number 2 (CNIM), which will be done independently.<br>If necessary reviewer number 3 (IL) will review if there are any disparities between the two initial reviews
**Narrative synthesis**<br>Details of what and how synthesis will be done	Narrative synthesis will be done alongside any meta-analysis and will be carried out using a framework which consists of four elements;<br>1. Developing a theory of how the method works, why and for whom<br>2. Developing a preliminary synthesis of findings of included studies<br>3. Exploring relationships within and between studies<br>4. Assessing the robustness of the synthesis

6. Presentation of results	
**Additional material**<br>Summary tables, flowcharts, etc, to be included in the final paper	Flow chart of whole process<br>Protocol<br>Data extraction form and tables<br>Forest plots of studies included in the final review
**Outputs from review**<br>Papers and target journals, conference presentations, reports, etc	X1 paper in high quality respiratory journal<br>Conference presentations at BTS<br>Report and presentation to fellowship

7. **Timeline for review**<br>(aim to complete each stage of the review)	
Protocol	Two months
Literature searching	Two months
Quality appraisal	Two months
Data extraction	Two months
Synthesis	Two months
Writing up	Two months

Please send your completed protocol to anca.pop@umfcd.ro

Anca Pop

Search strategy

1. Search queries - Keywords

(1) colorectal cancer AND NDRG4 AND early detection ;

(2) colorectal cancer AND BMP3 AND early detection

(3) colorectal cancer AND SEPT9 AND early detection or

(4) colorectal cancer AND SDC2 AND early detection .

(5) colorectal cancer AND epigenetics AND biomarkers or

(6) colorectal cancer AND DNA methylation biomarkers .

(7) early-stage colorectal cancer AND DNA methylation AND stool

(8) early-stage colorectal cancer AND DNA methylation AND blood

(9) early-stage colorectal cancer AND DNA methylation AND tissue

(10) early-stage colorectal cancer AND DNA methylation AND biopsy

(11) early detection of colorectal cancer AND DNA methylation

2. Eligibility

Inclusion criteria:

(1) DNA methylation marker specific for precancerous lesions and/or CRC Stage 0, I or stage II or

(2) significant difference between early-stage vs. advanced stage of CRC,

(3) biomarker performance assessment (e.g., sensitivity, specificity),

(4) a wide variety of probes and specimens,

(5) any method for methylation status determination.

Exclusion criteria:

1. Off topic research
    1.1. no screening objective
    1.2. other cancer types,
    1.3. other epigenetic alteration,
    1.4. other CRC stages (Advanced CRC),
    1.5. no DNA methylation biomarker,
    1.6. an analytical l approach
    1.7. treatment evaluation or
    1.8. no clinical or no experimental data
2. Study type: MA, SysR, Rev
3. Publication types:
    3.1. doctoral theses,
    3.2. position statements,
    3.3. conference abstracts,
    3.4. editorials,

3.5. reviews (SR),
3.6. meta-analyses (MA), or
3.7. study protocols.

3. Databases
    1. Web of Science
    2. Pubmed
    3. Scopus
    4. ProQuest

4. Period – 2017-2021 (five years)
5. Languages -all
6. Study types – RCT, CT, CCS, CC
7. Study subjects
    - Human
    - Culture Cells
    - And, if any
    - Vegetal cells
    - Animals

8. Research period – 10 07 2021-28.08.2021
9. Risk of Bias – method (AA)
10. Data search team - IL, CBI, SAA
11. Peer review – ALP
