# Supplementary material for: Promising Epigenetic Biomarkers for the Early Detection of Colorectal Cancer: A Systematic Review
Source: Cancers (Basel). 2021 Oct 2;13(19):4965. doi: 10.3390/cancers13194965 (PMC8508438; doi:10.3390/cancers13194965)
Supplement: Supplementary file 1 [file cancers-13-04965-s001.zip › Supplementary Tables S1-S4.pdf]

Supplementary Table S1 S2 S3 S4 S5

Sp.	Sensitivity (%)	Specificity (%)	Ref.
**Tissue**	83.1 (103/124) C**arcinoma**<br>56 (61/109) **AD**	90 (37/41)	[33]
**Stool**	81.1 (159/196) **CRC**<br>58.2 (71/122) **AD**	93.3 (167/179)	
**Tissue**	Just methylation status:<br>100 (18/18) C**arcinoma**<br>90.6 (29/32) **AD**<br>94.1 (16/17) **HP**<br>0 (0/5) **N**	-	[34]
**Stool**	83.3 (10/12) I<br>88.2 (15/17) II<br>90.0 (9/10) III<br>100 (11/11) IV	90.9 (20/22)	
**Tissue**	Just methylation status:<br>100 (2/2) **AA**<br>88.9 (8/9) **TA**<br>100 (3/3) **HP**<br>0 (0/5) **N**	-	[35]
**Bowel lavage**	80 **CRC**<br>64.7 **VA+HGD**	88.90	
**Stool**	100 (3/3) 0<br>85.5 (47/55) I<br>91.4 (64/70) II<br>89.6 (86/96) III<br>100 (21/21) IV<br>89.1 **early stage**	90.2	[36]
**Stool**	87 (134/154) I+II<br>42.1 **AA** (16/38)<br>79.9 **ACN** (371/397)<br>83.8 (301/359) **CRC**	98 (14/713)	[37]
**Stool**	77.4	88	[38]

Table S1. SDC2 performance in multiple studies. Sp. , specimen, AD, adenoma; CRC, colorectal cancer; TA, tubular adenoma; VA, villous adenoma; AA, advanced adenoma; HGD, high grade dysplasia; N, normal: HP, hyperplastic polyps; ACN, advanced colorectal neoplasia.

Screening method	Sensitivity (%)		Specificity (%)	AUC	Ref.
**mSEPT9**	61.8		89.6	0.757	[41]
**FOBT**	61.4		70.3	0.658	
**mSEPT9+FOBT**	**84.1**		**62.2**	**0.807**	
**CEA**	35.0		62.6	0.485	
**Ca-199**	17.9		55.7	0.353	
	CRC	A+P		-	[43]
**mSEPT9**	73	17.1	94.5		
**FOBT**	58.7	12.2	91.9		
**CEA vs mSEPT9**	NAA<br>22.0 vs 26.5<br>AA<br>27.5 vs 23<br>stage I<br>26.3 vs 52.6<br>stage II<br>53.3 vs 84.8<br>stage III<br>40.6 vs 78.8<br>stage IV<br>100.0 vs 100.0				[44]

Table S2. mSEPT9 performance comparison with other screening methods. A, adenoma; P, pol; SEPT9, Septin 9; FOBT, Fecal occult blood testing; CEA, Carcinoembryonic Antigen; Ca-199, Carbohydrate antigen 19-9.

Sp.	Sensitivity (%)				Specificity (%)		Ref.
**Tissue**	Just methylation profile:<br>mSEPT9 94.7% (18/19) mSDC2 100.0% (19/19) cancer tissues vs their paired adjacent paracancerous tissues				-		
**Serum***	**mSEPT9**<br>38.5 stage I (5/13)<br>81.6 stage II (40/49)<br>69.2 stage III (27/39)<br>100 stage IV (7/7)	**mSDC2**<br>53.9 stage I (7/13)<br>67.4 stage II (33/49)<br>79.5 stage III (31/39)<br>85.7 stage IV (6/7)	***ColoDefense***<br>69.2 stage I (9/13)<br>85.7 stage II (42/49)<br>89.7 stage III (35/39),<br>100 stage IV (7/7)		**mSEPT9**<br>95.6<br>**mSDC2**<br>95.6<br>***ColoDefense***<br>92.1		[48]
**Tissue**	Just methylation profile:<br>mSEPT9 92.5% (37/40) mSDC2 97.5% (39/40) cancer tissues vs their paired adjacent paracancerous tissues				-		[49]
**Plasma**	**mSEPT9**<br>65.0 stage I (13/20)<br>84.0 stage II (42/50),<br>86.8 stage III (33/38)<br>100 stage IV (4/4)<br>80.0 unknown stage (4/5)	**mSDC2**<br>55.0 stage I (11/20)<br>74.0 stage II (37/50)<br>65.8 stage III (25/38)<br>100 stage IV (4/4)<br>80.0 unknown stage (4/5)	***ColoDefense***<br>80.0 stage I (16/20)<br>90.0 stage II (45/50)<br>89.5 stage III (34/38)<br>100 stage IV (4/4)<br>100 unknown stage (5/5)	**mSEPT9**<br>CRC 82.1<br>**mSDC2**<br>CRC 69.2<br>***ColoDefense***<br>CRC 88.9<br>47.8 AA (11/23),<br>16.7 NAA (7/42),<br>27.6 HP (8/29),<br>42.9 MMD (3/7)	**mSEPT9**<br>CRC 95.8<br>**mSDC2**<br>CRC 95.8<br>***ColoDefense***<br>CRC 92.8	**mSEPT9**<br>SP 92.6<br>**mSDC2**<br>SP 93.4<br>***ColoDefense***<br>SP 87.7	
**Stool**	Training set<br>**mSEPT9**<br>4.6 control<br>50.0 AA<br>100 stage 0 CRC (1/1)<br>63.6 stage I CRC (7/11)<br>93.3 stage II CRC (14/15)<br>77.8 stage III CRC (14/18)<br>75.0 stage IV CRC (3/4)<br>66.7 unknown stage CRC (4/6)	Training set<br>**mSDC2**<br>6.2 control<br>33.3 AA<br>100.0 stage 0 CRC (1/1)<br>72.7 stage I CRC (8/11)<br>100.0 stage II CRC (15/15)<br>83.3%stage III CRC (15/18)<br>50.0 stage IV CRC (2/4)<br>83.3 unknown stage CRC (5/6) (5/6)	Training set<br>**ColoDefense**<br>9.2 control<br>66.7 AA<br>100 stage 0 CRC (1/1)<br>81.8 stage I CRC (9/11)<br>100.0 stage II CRC (15/15)<br>88.9 stage III CRC (16/18)<br>75.0 stage IV CRC (3/4)<br>83.3 unknown stage CRC (5/6)	Validation set<br>**mSEPT9**<br>50.0 AA<br>82.1 CRC<br>**mSDC2**<br>66.7 AA<br>87.2 CRC<br>**ColoDefense**<br>66.7 AA<br>92.3 CRC	**mSEPT9**<br>96.6<br>**mSDC2**<br>96.6<br>**ColoDefense**<br>93.2		[50]

Table S3. SEPT9 and SDC2 (Colodefense® test) performance in multiple studies. *Remained after biochemical test, Sp., specimen, SEPT9, septin 9; SDC2, syndecan-2; CRC, colorectal cancer; AA, advanced adenoma; NAA, non-advanced adenoma, HP, hyperplastic polyps; MMD, dysplasia of mild and moderate degrees.

mGenes	AUC	Sensitivity	Specificity	Ref.
EDNRB<br>Location 1<br>Location 2<br>Location 3<br>Location 4<br><br>KISS1	 0.949 (0.904–0.994)<br>0.931 (0.878–0.984)<br>0.858 (0.783–0.934)<br>0.887 (0.819–0.954<br><br>0.644 (0.530–0.759)	 **91.1%**<br>**91.1%**<br>**75.6%**<br>**80.0%**<br><br>**57.8%**	 **88.9%**<br>**80.0%**<br>**77.8%**<br>**84.4%**<br><br>**71.1%**	*[81]*
CTCF_33<br>CTCF_55<br>CTCF_94<br>CTCF_113<br>CTCF_13	**Stage I (N=39)**<br>CTCF_33, AUC: 0.9<br>CTFC_55, AUC: 1<br>CTCF_94, AUC: 1<br>CTCF_113, AUC: 1<br>CTCF_13, AUC: 1<br><br>**Stage II (N=101)**<br>CTCF_33, AUC: 0.9<br>CTFC_55, AUC: 1<br>CTCF_94, AUC: 0.9<br>CTCF_113, AUC: 1<br>CTCF_13, AUC: 1<br><br>**Stage III (N=47)**<br>CTCF_33, AUC: 0.9<br>CTFC_55, AUC: 0.9<br>CTCF_94, AUC: 0.9<br>CTCF_113, AUC; 0.9<br>CTCF_13, AUC: 1<br><br>**AD: (N=108)**<br>CTCF_33, AUC: 0.93<br>CTFC_55, AUC: 0.93<br>CTCF_94, AUC: 0.92<br>CTCF_113, AUC: 0.92<br>CTCF_13, AUC: 0.93	**CTCF_33:**<br>AD: 80.51%<br>Stage I: 67.47%<br>Stage II: 57.59%<br>Stage III: 65.22%<br><br>**CTCF_55**<br>AD:73.52%<br>Stage I: 95.13%<br>Stage II: 88.87%<br>Stage III: 80.77%<br><br>**CTCF_94**<br>AD: 79.44%<br>Stage I: 83.59%<br>Stage II:74.46%<br>Stage III:74.04%<br><br>**CTCF_113**<br>AD: 82.62%<br>Stage I: 92.92%<br>Stage II: 89.19%<br>Stage III:89.36%<br><br>**CTCF_13**<br>AD: 79.79%<br>Stage I: 96.84%<br>Stage II: 88.86%<br>Stage III:87.66%	**NA**	*[92]*
TWIST1	**NA**	**NNA**: 36.0%<br>AA: 30.0%<br>CRC: 44.4%	**92%**	*[74]*
		**NAA**: 35.0%<br>AA: 68.5%<br>CRC: 95.6%	**80.3%**	*[75]*
T-UCRs<br>(Uc160, Uc283<br>and Uc346)	**AC**<br>**Uc160:** 0.628<br>**Uc283:** 0.529<br>**Uc346:** 0.566<br><br>**AD or AC**<br>**Uc160:** 0.554<br>**Uc283**:0.511<br>**Uc346**: 0.562	**AC**<br>**Uc160:** 35%<br>**Uc283:** 12.5%<br>**Uc346:** 22.5%<br><br>**AD or AC**<br>**Uc160:** 20.8%<br>**Uc283:** 9.4%<br>**Uc346:** 17,1%	**AC**<br>**Uc160: 89%**<br>**Uc283: 92.7%**<br>**Uc346: 77.5%**<br><br>**AD or AC**<br>**Uc160: 88.7%**<br>**Uc283:92.7%**<br>**Uc346:94.3%**	*[82]*

ZNF331	**NA**	**71%**	**98%**	*[83]*
MPPED2	**0.89**	**80.40%**	**97.80%**	*[93]*
SFRP2	**0.82**	**CRC** **AA:** 42.9%<br>Stage I: 46.2%<br>Stage II: 74.1%<br>Stage III: 70.6%<br>Stage IV: 100%	**87.30%**	*[84]*
ADHFE1	**0.97**	96%	95%	*[85]*
DZIP3	**0.833**	**Early stage CRC**: 76%<br>**All stage CRC**: 72%	**Early stage CRC**: 77%<br>**All stage CRC**: 72%	*[13]*
Alpha1-Antitrypsin	**0.94**	**84%**	**100%**	*[91]*
CLIP4	**0.96**	**CRC**: 90.3% (84.2%–94.3%)<br>**AA**: 78.3% (55.8%–91.7%)	**88.4% (79.8%-93.8%)**	*[72]*
KCNQ5<br>C9orf50	**C9orf50+KCNQ5**: 0.88<br>**C9orf50**: 0.94<br>**KCNQ5**: 0.88	**C9orf50+KCNQ5:** 76.1%<br>**C9orf50**: 85.9%<br>**KCNQ5**: 77.3%	**C9orf50+KCNQ5**: 90.8%<br>**C9orf50**: 95.0%<br>**KCNQ5**: 91.5%	*[80]*
CfDNA	**0.91**	**84.60%**	**86.60%**	*[86]*
CtDNAmethylaion markers	**NA**	**88.60%**	**89.30%**	*[87]*
5hmC	**NA**	**84%**	**94%**	*[88]*
GDNF<br>HAND2<br>SLC35F3<br>SNAP91<br>SORCS1<br><br>JAM3	**GDNF**: 0.726<br>**HAND2**: 0.722<br>**SLC35F3**: 0.736<br>**SNAP91**: 0.799<br>**SORCS1**: 0.707<br><br>**JAM3**: 1	**GDNF**: 41.9%<br>**HAND2:** 32.6%<br>**SLC35F3**: 39.5%<br>**SNAP91**: 46.5%<br>**SORCS1**:41.9%<br><br>**JAM3**: NA	**98.0%**<br><br><br><br><br><br>**JAM3:** NA	*[89]*<br><br><br><br><br>*[90]*
KCNJ12<br>ZNF132	**0.799**<br>**0.764**	**62.4%**<br>**56.3%**	**88.7%**<br>**97.2%**	*[79]*

Table S4. **Potential biomarkers performance in multiple studies.** CRC, colorectal cancer; AA, advanced adenoma; AC, Adenocarcinma; AD, adenoma; ADHFE1, AlcoholDehydrogenase Iron Containing 1; C9orf50,Chromosome 9 Open Reading Frame 50; CLIP4, CAP-Gly Domain Containing Linker Protein Family Member 4;

cf-DNA, cell- free DNA; CTCF, CCCTC-Binding Factor; DZIP3, DAZ Interacting Zinc Finger Protein 3; EDNRB, Endothelin Receptor Type B; GDNF, Glial Cell Derived Neurotrophic Factor; HAND2, Heart And Neural Crest Derivatives Expressed 2; KCNQ5, Potassium Voltage-Gated Channel Subfamily Q Member 5; KCNJ12, Potassium Inwardly Rectifying Channel Subfamily J Member 12; KISS1, KiSS-1 Metastasis Suppressor; MPPED2, Metallophosphoesterase Domain Containing 2; NAA, Non-advanced adenoma; SFRP2, Secreted Frizzled Related Protein 2; SLC35F3, Solute Carrier Family 35 Member F3; SNAP91, Synaptosome Associated Protein 91; SORCS1, Sortilin Related VPS10 Domain Containing Receptor 1;, 5hmC, 5-Hydroxymethylcytosine; T-URCs, Transcribed Ultraconserved Regions (T-UCRs), TWIST1, Twist Family BHLH Transcription Factor; ZNF132, Zinc Finger Protein 132; ZNF331, Zinc Finger Protein 331, JAM3, Junctional Adhesion Molecule 3
